# Alcohol Consumption among the Elderly Citizens in Hungary and Serbia—Comparative Assessment

**DOI:** 10.3390/ijerph17041289

**Published:** 2020-02-17

**Authors:** Natasa Mihailovic, Gergő József Szőllősi, Nemanja Rancic, Sándor János, Klára Boruzs, Attila Csaba Nagy, Yuriy Timofeyev, Viktorija Dragojevic-Simic, Marko Antunovic, Vladimir Reshetnikov, Róza Ádány, Mihajlo Jakovljevic

**Affiliations:** 1 Institute of Public Health Kragujevac, Department of Biostatistics and Informatics, Nikole Pašića 1, 34000 Kragujevac, Serbia; 2 Department of Preventive Medicine, Faculty of Public Health, University of Debrecen, H-4002 Debrecen, Hungary; 3 The Centre for Clinical Pharmacology, Medical Faculty, Military Medical Academy, University of Defence, Crnotravska 17, 11000 Belgrade, Serbia; 4 Department of Health Systems Management and Quality Management in Health Care, Faculty of Public Health, University of Debrecen, H-4002 Debrecen, Hungary; 5 National Research University Higher School of Economics, Shabolovka Ulitsa 26-28, 119049 Moscow, Russian; 6 National Poison Control Center, Medical Faculty, Military Medical Academy, University of Defence, Crnotravska 17, 11000 Belgrade, Serbia; 7 N.A. Semashko Department of Public Health and Healthcare, I.M. Sechenov the First Moscow State Medical University (Sechenov University), 119991 Moscow, Russia; 8 MTA-DE Public Health Research Group, Public Health Research Institute, University of Debrecen, H-4002 Debrecen, Hungary; 9 Department of Global Health Economics and Policy, Faculty of Medical Sciences, University of Kragujevac, 34000 Kragujevac, Serbia; 10 Institute of Comparative Economic Studies, Hosei University, Tokyo 194-0298, Japan

**Keywords:** alcohol consumption, socio-demographic factors, Hungary, Serbia

## Abstract

Studies in the alcohol consumption area are mostly related to the (ab)use of alcohol in young people. However, today, a growing number of researchers are emphasizing the clinical and public health significance of alcohol consumption in the elderly. In the WHO reports, harmful alcohol consumption is responsible for 5.3% of the global burden of the disease. The aim of this study was to investigate the prevalence of alcohol consumption among men and women aged 55 and over in Serbia and Hungary, leveraging data from the 2013 Serbian National Health Survey and from the 2014 Hungarian National Health Survey. Respondents aged 55 and over were analysed based on logistic multivariate models. The prevalence of alcohol consumption was 41.5% and 62.5% in Serbia and Hungary, respectively. It was higher among men in both countries, but among women, it was significantly higher in Hungary than in Serbia. The statistically significant predictors affecting alcohol consumption in Serbia included age, education, well-being index, long-term disease and overall health status, with marital status being an additional factor among men. In Hungary, education and long-term disease affected alcohol consumption in both sexes, while age and employment were additional factors among women. In both countries for both sexes, younger age, more significantly than primary education and good health, was associated with a higher likelihood of alcohol consumption.

## 1. Introduction

Alcoholism is the most widespread addiction disease. Alcohol consumption is associated with more than 200 illnesses/health disorders, the most frequent of which include DSM–IV alcohol dependence, liver cirrhosis, cancers and injuries [1]. According to the World Health Organization (WHO), three million people die annually from the effects of harmful alcohol consumption worldwide while according to the disability-adjusted life years (DALYs), 5.3% of the global burden of disease was attributable to alcohol consumption in 2016 [2]. Alcohol addiction is defined not only by the amount but also by the frequency of alcohol consumption. Eurostat data show that in 2014, three out of four EU citizens consumed alcohol, while one in 11 EU citizens consumed alcohol daily [3].

In 2006, the European Commission started “An EU strategy to support Member States in reducing alcohol-related harm” (COM (2006) 625 final), aiming to reduce the alcohol consumption by raising people’s awareness of its harmfulness. Thus, alcohol-related deaths, total alcohol consumption (recorded and unrecorded) and hazardous alcohol consumption were listed as some of the 88 European Core Health Indicators [4]. Moreover, in 2012, following the global strategy to reduce the harmful effects of alcohol, members of the WHO European Region endorsed “The European action plan to reduce the harmful use of alcohol 2012–2020” [5]. 

The total alcohol per capita (APC) is defined as the amount of alcohol per capita expressed in litres of pure alcohol consumed yearly. The APC trend analysis, from 2010 to 2016, shows that Hungary is following the European decreasing trend, changing from 12.1 to 11.4 litres of pure alcohol (with an average alcohol consumption of 19.1 litres and 4.5 litres for males and females respectively) [2]. At the same time, in 2016, in Serbia, there was a slight decrease in APC from 11.7 to 11.1 litres of pure alcohol (with an average alcohol consumption of 18.5 litres and 4.1 litres for males and females respectively). On the one hand, the prevalence of heavy episodic drinking (consumed at least 60 g or more of pure alcohol on at least one occasion in the past 30 days) among the citizens aged 15 and over, were reported as 33.5% and 29% for Hungary and Serbia, respectively. On the other hand, in 2016, 40% of Serbian respondents and 33.4% of Hungarian respondents did not consume alcohol in the previous 12 months at all. The prevalence of alcohol use disorders in Hungary was significantly higher than the average of the WHO European Region—21.2% versus 8.8%, respectively, and so was the prevalence of alcohol dependence, which was 9.4% in Hungary versus 3.7% in Europe. At the same time, the prevalence of alcohol use disorders in Serbia was 5.9%, and the prevalence of alcohol dependence was 3.4%. Among those who drink alcohol, men were more likely in comparison to women to report having an episode of heavy drinking at least once a month (the percentage for men was 2.3 times higher than for women; 28.3% for men and 12.3% for women) [3]. However, unlike Hungary, there is no established National Monitoring System in Serbia so the issue of proper record keeping remains present and caution is necessary when interpreting data [6].

Until recently, research studies were mostly related to the (ab)use of alcohol in young people [7,8]. The European School Survey Project on Alcohol and Other Drugs represents the most complete research on alcohol abuse among high school students, which has been conducted in most European countries periodically since 1995 [7].

However, today, a growing number of researchers are emphasizing the clinical and public health significance of alcohol consumption in the elderly, both in developed and in developing countries [9,10]. Although there are different alcohol use patterns among European countries and the prevalence of alcohol use disorders, alcohol consumption patterns in the elderly are linked not only to certain cultural patterns of behaviour and aging-related health conditions, but also to the need for relaxation [11,12,13]. On the relevant medical databases, at this moment, few studies are available on alcohol consumption among older people, but the data are very different and there is no systematic data analysis on this topic [14,15,16,17,18].

The aim of this study was to investigate the prevalence and factors related to alcohol consumption among men and women aged 55 and over in Serbia and Hungary, analysing the data from the latest published National Health Surveys.

## 2. The Database from Both Countries

The second wave of the European Health Interview Survey (EHIS 2) took place in Serbia in 2013 and in Hungary in 2014. Then, the cross-sectional study data were merged into a single common database which was further analysed.

### 2.1. Sample from Serbia

The 2013 Serbian National Health Survey included respondents aged 15 and over. The total number of respondents was 14,623, out of whom 6421 were adults aged 55 and over. The analysed respondents were divided into regions and each region was further divided by type into urban (N = 3513; 54.7%) and rural areas (N = 2908; 45.3%). The residents of Kosovo and Metohija, as well as residents of special institutions (retirement homes, prisons and psychiatric clinics) were excluded from the survey. The question “How often did you consume alcohol in the previous twelve months?” had a response rate of 85.8% (N = 5510). Nonresponse was more common among females and in those aged 75 and over.

### 2.2. Sample from Hungary

The 2014 Hungarian National Health Survey included respondents aged 15 and over. The total number of respondents was 5815, of whom 2190 were adults aged 55 and over. The analysed respondents were divided into regions and each region was further divided by type, into urban (N = 1535; 70.1%) and rural areas (N = 655; 29.9%). Similarly, the residents of special institutions were excluded from the survey. The question “How often did you consume alcohol in the previous twelve months?” had a response rate of 99.8% (N = 2185); only five participants refused to answer.

### 2.3. Survey Data Description

The main survey question was: “How often did you consume alcohol in the previous twelve months (beer, wine, cocktails, homemade alcoholic drinks)?” The possible answers were: “1–2 days per week”, “2–3 days per week”, “3–4 days per week”, “5–6 days per week”, “every day or almost every day”, “once a month”, “less than once a month”, “not once in the past 12 months because I do not drink alcohol anymore/I have never drunk alcohol in my entire life” (dependent variable). For the purpose of statistical analysis, regrouping of the answers was done and the respondents were divided into two groups: those who answered never or not once in the past 12 months were classified as alcohol non-users, while everyone else was classified as an alcohol user. The independent variables included socio-demographic characteristics and general health status. Socio-demographic characteristics included: sex; age divided in age groups 55–64, 65–74 and 75 and over; education (primary or less, secondary, and tertiary); employment (work; retired; and unemployed, which included and unable to work); living area (urban or rural area); marital status (married and not married, which included the categories: divorced, never married, widowed); well-being index as a complex index created by combining multiple simple indices that relate to the possession of different household assets (for these research categories, second, third and fourth were put under one named middle and categories of responses were: poor, middle and rich class); and long-term disease, which is equal to one if a disease that have lasted 6 months or longer takes place. Variable overall health status referred self-assessment of health of respondents. Categories of responses were: “very good “good”, “not good”, “not bad”, “bad”, and “very bad”. For the purpose of this research, the categories “good”, “not good”, “not bad” and “bad” were put under one name middle. Thus, overall health status could be: “very good”, “middle” and “very bad”.

The total number of respondents was 8611. The average response rate to the main survey question was 89.4% (85.8% in Serbia and 99.8% in Hungary).

### 2.4. Statistical Analysis

Categorical variables were presented as frequency and percentage. The binary logistic regression was applied as a multivariate statistical method. Alcohol consumption was the dependent variable while the independent ones included gender, age, education, employment, place of living, marital status, well-being index, long-term illness and overall health status. The marital status variable was transformed so that the responses “a married couple” and “a cohabiting couple” were classified as “living with a partner”, and all other answers were classified into living without a partner (divorced; having lived with a partner which ended with a breakup; never married; never lived with a partner; widow/widower; lived with a partner which ended with the death of the partner). In addition, the well-being index variable was transformed into three categories: poor, middle class (which contained categories: second, third and fourth answer) and rich. The overall health status variable was transformed into very bad, middle (bad, fair and good) and very good.

Odds ratios and 95% confidence intervals were defined. All the analyses were performed using IBM SPSS software, version 19 (IBM, Armonk, NY, USA).

## 3. Results

**Figure 1:**
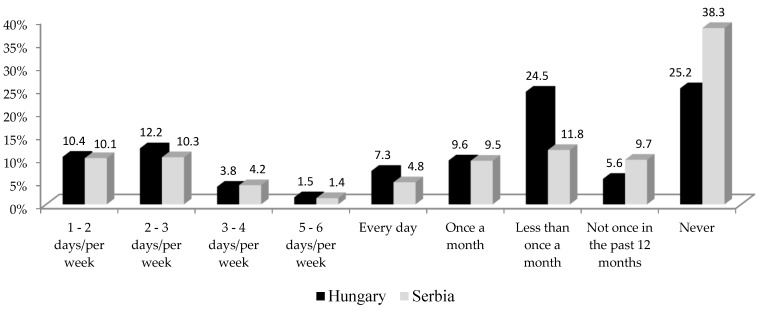
The prevalence of alcohol consumption among older adults in Serbia and Hungary.

In Serbia, the prevalence of alcohol consumption was 41.5% while in Hungary, it was 62.5% (Figure 1). The gender analysis showed that the prevalence of alcohol use among men was higher than in women in both study populations (men vs. women; Serbia: 63.2% vs. 23.4%, Chi-square test = 891.86, df = 1, *p* < 0.01; Hungary: 79.8% vs. 50.1%, Chi-square test = 199.28, df = 1, *p* < 0.01). At the same time, the prevalence of alcohol consumption in both genders in Hungary was significantly higher (Hungary vs. Serbia; men: 79.8% vs. 63.2%, Chi-square test = 83.22, df = 1, *p* < 0.01; women: 50.1% vs. 23.4%, Chi-square test = 296.15, df = 1, *p* < 0.01). The prevalence of alcohol consumption, classified by socio-demographic characteristics and health status of the respondents, was higher among the male population in both countries. However, alcohol consumption is increasing with the education level in both countries and both sexes (Table 1).

The analysis of alcohol consumption predictors among Serbian women demonstrates that with age, alcohol consumption decreases. Namely, women aged 65–74, as well as those aged 75 and over, consumed significantly less alcohol compared to women aged 55-64 (OR = 0.65, 95% CI = 0.5–0.85, or OR = 0.75. 95% CI = 0.57–0.99). As the level of education increased, so did the likelihood of consuming alcohol: women with secondary education were almost four times more likely (OR = 3.95, 95% CI = 2.8–5.57), and those with tertiary education were 2.5 times (OR = 2.45, 95% CI = 1.78–3.36) more likely to consume alcohol compared to women with primary education or lower. According to the well-being index, women who were classified in the middle (OR = 1.57, 95% CI = 1.1–2.24) and rich class (OR = 1.5, 95% CI = 1.15–1.95) were significantly more likely to consume alcohol compared to those who were classified as poor. Moreover, women who were not diagnosed with a long-term disease were almost twice as likely to consume alcohol compared to those who were (OR = 1.77, 95% CI = 1.45–2.15). Similarly, women who rated their own health as poor (OR = 0.48, 95% CI = 0.3–0.76), or medium (OR = 0.28, 95% CI = 0.14–0.55) were less likely to consume alcohol compared to women who rated their health as very good. Statistically insignificant predictors included employment, place of living, and marital status (Table 2).

The analysis of predictors of alcohol consumption among Hungarian women shows that women aged 65–74 were significantly less likely (OR = 0.6, 95% CI = 0.41–0.86) to drink alcohol compared to women aged 55–64. Similarly to the results obtained from women in Serbia, women with higher education in Hungary were more likely to consume alcohol, women with secondary education were two times more likely (OR = 1.96, 95% CI = 1.33–2.87), and those with tertiary education were 1.5 times (OR = 1.52, 95% CI = 1.07–2.16) more likely to consume alcohol compared to those with primary education or lower. Alcohol is consumed less by unemployed (OR = 0.49, 95% CI = 0.31–0.77) and retired women (OR = 0.42, 95% CI = 0.26–0.69). Women who were not diagnosed with a long-term disease were 1.5 times more likely to consume alcohol compared to women who were (OR = 1.52, 95% CI = 1.14–2.03). Statistically insignificant predictors included place of living, marital status, well-being index and overall health status (Table 2).

The analysis of alcohol consumption predictors among Serbian men showed that as among women, the likelihood of alcohol consumption decreased with age. Men with secondary education were 1.5 times more likely to consume alcohol (OR = 1.49, 95% CI = 1.06–2.09) compared to men with primary education or lower. In addition, married (OR = 0.75, 95% CI = 0.61–0.92) and rich men (OR = 0.75, 95% CI = 0.61–0.92) were more likely to consume alcohol than unmarried and poor ones. Health status was a factor significantly affecting the likelihood of consuming alcohol so that the males who had not been diagnosed with a long-term disease, were almost two times more likely to consume alcohol (OR = 1.88, 95% CI = 1.56–2.27). Men who self-rated their own health as middle (OR = 0.75, 95% CI = 0.61–0.92) and very poor (OR = 0.75, 95% CI = 0.61–0.92), consumed alcohol almost two times less frequently compared those who rated their overall health status as very good. Work status and place of living were not statistically significant (Table 3).

The analysis of alcohol consumption predictors among Hungarian men showed that only two variables had a statistically significant contribution to the model: education and long-term disease. Namely, men with secondary education were three times more likely (OR = 3.16, 95% CI = 1.75–5.7), and those with tertiary education were almost two times more likely (OR = 1.84, 95% CI = 1.1–3.07) to consume alcohol compared to men with a primary education or lower. In addition, healthy men consumed alcohol 1.7 times more often (OR = 1.7, 95% CI = 1.12–2.59) compared to men diagnosed with a long-term disease (Table 3).

## 4. Discussion

The results of National Health Surveys in several countries can be compared due to their similar research methodologies. This fact is very important, especially when investigating health disorders for which there are no registers established, as in case of alcoholism in Serbia [19]. Alcoholism, as a disease of addiction, affects not only the individual and his family, but also the society as a whole. Namely, the abuse of alcohol has a large socio-medical impact because, apart from the need for long-term treatment, hospitalization and rehabilitation, alcohol consumption is often associated with traffic accidents, injuries, fights, violence and reduced productivity, all of which affect the health and socio-economic condition of the individual, family and society in general [20]. Illegal production and consumption of alcohol is a particular problem. Difference in alcohol-attributable mortality in Central and Eastern Europe and in Western Europe can also be caused by unrecorded alcohol consumption, such as home-made fruit spirits, which, in addition to ethanol, have higher concentrations of aliphatic alcohols with high hepatotoxicity [21]. Some studies indicate that more than a quarter of all consumed alcohol is distributed and sold outside formal channels [22]. Therefore, a survey conducted in Hungary indicated that restrictions on supply and sale of alcohol from illicit sources are needed to reduce alcohol-attributable mortality significantly [23].

The trend of increasing morbidity and mortality in the elderly caused by the constant consumption of alcohol has become more obvious in recent years [17].

Alcohol consumption in both Serbia and Hungary is treated as socially acceptable behaviour, most often related to certain social events and patterns of behaviour which encourage individuals to consume alcohol. For both men and women, the prevalence of alcohol consumption in Hungary is higher than in Serbia. This difference can be related to the difference in the economic development and standard of living between the two countries [24]. Namely, in 2018, Hungary, being a member of the European Union, had a gross domestic product per capita of 30,673,080 PPP (current international USD). At the same time, Serbia, which was in the process of joining the European Union, had a gross domestic product per capita, almost two times lower: 17,404,275 PPP (current international USD) [25].

Similarly to previous research studies, this study showed that men in Serbia and Hungary are more likely to consume alcohol compared to women, which can be related not only to the cultural heritage and the generally accepted model of behaviour, but also to the greater economic power of men. Evidence suggests that the gender gap in socio-economic status is significant between the two countries [26,27,28,29,30].

The fact that more educated respondents, both males and females, were more likely to consume alcohol was also confirmed in a survey conducted in 15 countries in Europe and South America [31]. Unlike men, whose place of residence has no influence on alcohol consumption; women living in urban areas are more likely to consume alcohol compared to women from rural areas, which is related to the higher employment rates and better socio-economic status of these women [32]. In Serbia, married men are more likely to consume alcohol, unlike the respondents in the UK where alcohol is consumed more often by older people living alone [33]. In Serbia, like in other countries, the well-being index is directly linked to alcohol consumption, thus, the poorest women and men are the least likely to consume alcohol [34,35,36,37], although less frequent drinking does not mean necessarily less harmful drinking. Findings have indicated that people with higher SES may consume similar or greater amounts of alcohol compared to people with lower socio-economic status (SES), although the latter group seems to bear a disproportionate burden of negative alcohol-related consequences [38]. This has been referred to as the “alcohol harm paradox”, which includes socio-economic differences in patterns of drinking, higher prevalence of other health harming behaviours (smoking, unhealthy nutrition, and lack of exercise), poorer quality of health care and support services, weaker social support networks, underestimation of consumption levels and alcohol-related harms, and the experience of Adverse Childhood Experiences in people with lower SES [39].

There is a direct correlation between alcohol consumption and a shorter life expectancy. Namely, as the number of people aged 65 and over increases, the prevalence of long-term illness and healthcare expenditures increases. Similarly to the results of our study, a survey conducted in the United States showed that two thirds of respondents aged 65 and over with chronic medical conditions did not consume alcohol [37]. Life experience had the greatest impact on reduced alcohol use in the elderly, together with the presence of long-term disease and overall health status.

## 5. Conclusions

This was the first study to compare the consumption and factors affecting alcohol consumption among people aged 55 and over in two neighbouring countries, namely, Hungary and Serbia, one of which is an EU member state and the other which is in the process of joining.

The limitations of this study are as follows. The frequency of alcohol consumption, as well as the quantity and type of alcoholic beverages, were not analysed. In addition, the study did not include people living in specialised institutions, such as nursing homes.

The main findings can be summarised as follows. The prevalence of alcohol consumption was higher in Hungary, both among men and women. Younger age, higher education, the absence of long-term disease, being retired or unemployed were associated with a higher likelihood of alcohol consumption among women, while higher education and the absence of long-term disease were associated with a higher likelihood of alcohol consumption among men. In Serbia, for both men and women, a higher likelihood of alcohol consumption was associated with younger age, higher education, better socio-economic status, the absence of long-term disease, and good overall health.

## Figures and Tables

**Table 1:** The prevalence of alcohol consumption among older adults in Serbia and Hungary.

Variables	Men					Women					Serbia	Hungary
	Serbia		Hungary		*p* *	Serbia		Hungary		*p* *	Men/Women	
	Total	Prevalence N (%)	Total	Prevalence N (%)		Total	Prevalence N (%)	Total	Prevalence N (%)		*p* *	*p* *
*Alcohol* Consumption **	2856	1584 (55.5)	913	726 (79.5)		3565	702 (19.7)	1277	639 (50)			
*Age*												
55–64	1216	842 (69.2)	452	378 (83.6)	>0.05	1384	392 (28.3)	519	308 (59.3)	<0.05	>0.05	>0.05
65–74	766	478 (62.4)	279	221 (79.2)		915	200 (21.9)	410	191 (46.6)			
75+	525	264 (50.3)	179	127 (70.9)		704	110 (15.6)	346	140 (40.5)			
*Education*												
Primary or less	757	424 (56)	183	126 (68.9)	<0.01	1621	252 (15.5)	498	198 (39.80	<0.05	<0.01	<0.01
Secondary	1462	953 (65.2)	531	429 (80.8)		1173	338 (28.8)	578	310 (53.6)			
Tertiary	288	207 (71.9)	196	171 (87.2)		209	112 (53.6)	199	131 (65.8)			
*Employment*												
Work	424	320 (75.5)	242	212 (87.6)	<0.01	668	150 (22.5)	197	135 (68.5)	>0.05	<0.01	<0.01
Retired	1695	1000 (59)	571	440 (77.1)		2027	492 (24.3)	952	455 (47.8)			
Unemployed	388	264 (68)	97	74 (76.3)		308	60 (19.5)	125	48 (38.4)			
*Living in*												
Urban areas	1339	869 (64.9)	646	519 (80.3)	<0.01	1712	482 (28.2)	885	465 (52.5)	>0.05	<0.01	>0.05
Rural areas	1168	715 (64.2)	264	207 (78.4)		1291	220 (17)	390	174 (44.6)			
*Marital status*												
Married	1983	1290 (65.1)	657	528 (80.4)	<0.01	1654	408 (24.7)	596	334 (56)	<0.05	<0.01	<0.01
Not married	524	294 (56.1)	253	198 (78.3)		1349	294 (21.8)	679	305 (44.9)			
*Well-being index*												
Poor	694	418 (60.2)	190	147 (77.4)	<0.05	855	136 (15.9)	468	197 (42.1)	<0.01	<0.01	<0.01
Middle class	1496	930 (62.2)	592	470 (79.4)		1790	417 (23.3)	688	364 (52.9)			
Rich	317	236 (74.4)	128	109 (85.2)		358	149 (41.6)	119	78 (65.5)			
*Long-term disease*												
Yes	1560	883 (56.6)	646	498 (77.1)	<0.01	2228	436 (19.6)	970	447 (46.1)	<0.05	<0.05	>0.05
No	947	701 (74)	264	228 (86.4)		767	265 (34.6)	304	191 (62.8)			
*Overall health status*												
Very good	146	110 (75.3)	40	33 (82.5)	<0.01	77	37 (48.1)	48	32 (66.7)	>0.05	>0.05	>0.05
Middle	2249	1428 (63.5)	806	646 (80.1)		2700	643 (23.8)	1116	571 (51.2)			
Very bad	110	44 (40)	64	47 (73.4)		226	22 (9.7)	111	36 (32.4)			

* Chi-squared test; Prevalence N (%) states for prevalence of alcohol consumption as a number (percentage); ** Data are missing for 349 men and 562 women from Serbia, and for three men and two women from Hungary.

**Table 2:** Binary logistic regression for alcohol consumption among older women in Serbia and Hungary.

Variables		Serbia			Hungary			
	B	Exp(B)	95%CI	*p*	B	Exp(B)	95%CI	*p*
*Age*								
55–64		1			1			
65–74	−0.43	0.65	0.5–0.85	<0.05	−0.52	0.6	0.41–0.86	<0.05
75+	−0.28	0.75	0.57–0.99	<0.05	−0.01	0.99	0.73–1.35	>0.05
*Education*								
Primary or less	1				1			
Secondary	1.37	3.95	2.8–5.57	<0.01	0.67	1.96	1.33–2.87	<0.05
Tertiary	0.89	2.45	1.78–3.36	<0.01	0.42	1.52	1.07–2.16	<0.05
*Employment*								
**Work**	1				1			
Retired	−0.19	0.83	0.58–1.18	>0.05	−0.87	0.42	0.26–0.69	<0.05
Unemployed	−0.25	0.78	0.56–1.08	>0.05	−0.72	0.49	0.31–0.77	<0.05
*Living in*								
Urban areas	1				1			
Rural areas	−0.21	0.81	0.65–1.02	>0.05	−0.17	0.84	0.65–1.09	>0.05
*Marital status*								
Married	1				1			
Not married	−0.03	0.97	0.8–1.18	>0.05	−0.23	0.79	0.6–1.04	>0.05
*Well-being index*								
Poor	1				1			
Middle class	0.45	1.57	1.1–2.24	<0.05	0.34	1.4	0.87–2.25	>0.05
Rich	0.41	1.5	1.15–1.95	<0.05	0.27	1.31	0.85–2.01	>0.05
*Long-term disease*								
Yes	1				1			
No	0.57	1.77	1.45–2.15	<0.01	0.42	1.52	1.14–2.03	<0.05
*Overall health status*								
Very good	1				1			
Middle	−1.27	0.28	0.14–0.55	<0.01	−0.41	0.66	0.3–1.45	>0.05
Very bad	−0.74	0.48	0.3–0.76	<0.05	−0.36	0.7	0.45–1.08	>0.05

**Table 3:** Binary logistic regression for alcohol consumption among older men in Serbia and Hungary.

Variables		Serbia			Hungary			
	B	Exp(B)	95%CI	*p*	B	Exp(B)	95%CI	*p*
*Age*								
55–64		1			1			
65–74	−0.45	0.64	0.49–0.83	<0.05	−0.5	0.61	0.35–1.04	>0.05
75+	−0.42	0.66	0.52–0.83	<0.01	−0.29	0.75	0.48-1.19	>0.05
*Education*								
Primary or less	1				1			
Secondary	0.4	1.49	1.06–2.09	<0.05	1.15	3.16	1.75–5.7	<0.01
Tertiary	0.17	1.18	0.88–1.6	>0.05	0.61	1.84	1.1–3.07	<0.05
*Employment*								
Work	1				1			
Retired	−0.18	0.84	0.61–1.16	>0.05	−0.58	0.56	0.29-1.08	>0.05
Unemployed	0.16	1.17	0.88–1.56	>0.05	−0.21	0.81	0.43-1.54	>0.05
*Living in*								
Urban areas	1				1			
Rural areas	−0.05	0.95	0.77–1.16	>0.05	0.1	1.11	0.76–1.62	>0.05
*Marital status*								
Married	1				1			
Not married	−0.29	0.75	0.61–0.92	<0.05	−0.06	0.94	0.62–1.41	>0.05
*Well-being index*								
Poor	1				1			
Middle class	0.28	1.32	0.92–1.89	>0.05	−0.24	0.79	0.39–1.61	>0.05
Rich	0.42	1.52	1.13–2.05	<0.05	−0.13	0.88	0.49–1.58	>0.05
*Long-term disease*								
Yes	1				1			
No	0.63	1.88	1.56–2.27	<0.01	0.53	1.7	1.12–2.59	<0.05
*Overall health status*								
Very good	1				1			
Middle	−0.77	0.46	0.26–80.82	<0.05	0.36	1.43	0.49–4.14	>0.05
Very bad	−0.65	0.52	0.35–0.78	<0.05	−0.13	0.88	0.48–1.61	>0.05
